# Risk factors for new bovine brucellosis infections in Colombian herds

**DOI:** 10.1186/s12917-019-1825-9

**Published:** 2019-03-07

**Authors:** Liliana Cárdenas, Mario Peña, Oscar Melo, Jordi Casal

**Affiliations:** 1 Animal Medicine and Health Department, Faculty of Veterinary Medicine, Campus of The Autonomous University of Barcelona (UAB), 08193 Bellaterra, Barcelona, Spain; 2 The Centre for Research into Animal Health (CReSA), Campus of The Autonomous University of Barcelona (UAB), 08193 Bellaterra, Barcelona, Spain; 3 Animal Health Department, Colombian Veterinary service, Instituto Colombiano Agropecuario-ICA, 11161 Bogotá, Colombia; 4 The Statistics Department, Faculty of Science, National University of Colombia, 11001 Bogotá, D.C Colombia

**Keywords:** *Brucella-free herds*, Bovine brucellosis infection, Colombia, Risk factors

## Abstract

**Background:**

Bovine brucellosis is a zoonotic disease that causes substantial economic losses and has a strong impact on public health. The main objective of this paper is to determine the risk factors for new infections of *Brucella abortus* on Colombian cattle farms previously certified as being free of brucellosis. A case-control study was conducted by comparing 98 cases (farms certified as brucellosis-free for three or more years but became infected) with 93 controls (farms that remained brucellosis-free during at least the previous three years). The farms were matched by herd size and geographical location (municipality). Information was obtained via a questionnaire completed by veterinary officers through a personal interview with the herd owners.

**Results:**

Two-thirds of the herds (67%) were dairy herds, 16% were beef herds, and 17% were dual-purpose (beef and milk) herds. After exploratory univariate analysis, all explanatory variables with a *p*-value of ≤0.20 were included in a logistic regression model using the forward stepwise method to select the model with the best goodness of fit. The significant risk factors were the replacement of animals from farms not certified as brucellosis-free compared to replacement from certified brucellosis-free farms (OR = 4.84, *p*-value < 0.001) and beef cattle farms compared to dairy cattle farms (OR = 3.61, *p*-value = 0.017). When herds with and without artificial insemination were compared, it was observed that farms that used natural breeding with bulls from non-certified herds had a higher risk than farms using artificial insemination (OR = 2.45, *p*-value = 0.037), but when the bulls came from brucellosis-free farms, farms with natural breeding were less affected (OR = 0.30, p-value = 0.004) than farms using artificial insemination, whether with frozen semen from certified brucellosis-free herds or fresh semen from uncontrolled herds. The latter is commonly sold to neighbouring farms.

**Conclusions:**

The government should make efforts to inform farmers about the risks involved in the introduction of semen and replacement heifers from farms that are not certified as brucellosis-free and to establish measures to control these practices.

**Electronic supplementary material:**

The online version of this article (10.1186/s12917-019-1825-9) contains supplementary material, which is available to authorized users.

## Background

Bovine brucellosis is an important zoonosis with worldwide distribution. The infection has been controlled or eliminated in most developed countries, but it remains endemic in Africa, Latin America and Asia [1].

Bovine brucellosis is a chronic, infectious disease caused by *Brucella abortus,* which has developed mechanisms to live intracellularly and is able to infect cattle for long periods of –time [2, 3]. Some animals are asymptomatic, having latent infection without exhibiting clinical signs, thus maintaining the disease in a herd [4, 5].

Cattle become infected after the ingestion of contaminated milk, food, water or grazing forage; close contact with infected animals; contact with uterine secretions or aborted foetuses; and through vertical and sexual transmission [6, 7]. The disease directly affects cattle and buffalo, which are the main reservoirs of *B. abortus*, although other domestic and wild species can also act as a reservoir [8, 9]. The main clinical signs are abortion, infertility, stillbirth or the birth of weak calves, along with epididymitis and orchitis in males [10, 11].

The introduction of cattle from infected herds or in contact with false-negative animals can contribute to introducing the infection into brucellosis-free farms [12]. Transmission within and between farms has been associated with different risk factors: the maintenance of positive animals in the herds, large farms, communal pastures, semi-intensive production systems and age (the infection is more prevalent in adults) [13]. Brucellosis is considered a complex disease due to its wide range of hosts and the variable signs at both the individual and population levels. Infected animals can remain infectious after their first abortion and spread the disease [14]. On the other hand, the disease can be misclassified as other reproductive diseases, which is one of the reasons that bovine brucellosis is generally underestimated at the farm level [14, 15]. Only *Brucella abortus* has been reported in Colombia. There are no studies concerning the epidemiology of brucellosis in the human population in Colombia, but it is assumed that in terms of human health, brucellosis is an under-diagnosed and under-notified disease [16].

The methods to control and eliminate brucellosis from a region are based on vaccination, controlling movements, and testing and removing serologically positive animals. Considerable effort and time are required to achieve disease-free status.

Control efforts against bovine brucellosis in Colombia are led through an official programme. This programme is voluntary, includes 15% of farms and is based on the identification of animals, compulsory vaccination of calves between 3 and 8 months of age (to make this vaccination compatible with further serological tests), control of animal movement, and testing and removal of positive animals older than 24 months (8 months in the case of males). The diagnosis of bovine brucellosis is made by serological serial testing with the Rose Bengal plate test and indirect ELISA and is complemented by competitive ELISA.

Blood sampling is performed by the veterinary service or by authorized veterinarians, and farms are classified as brucellosis-free if all animals are negative in two consecutive serological diagnostic tests. In 2016, 18,996 herds had achieved the status of officially brucellosis-free (4% of the 514,794 cattle farms present in the country).

In a previous study about bovine brucellosis in Colombia, heterogeneity was found in the control efforts and prevalence within the country [17]. In recent years, several farms classified as bovine brucellosis-free have become infected, in most cases without knowledge of the causes of these new infections. The aim of this study is to identify the risk factors associated with new infections of *Brucella abortus* detected between 2015 and 2016 on cattle farms previously certified as being free of the disease. A secondary objective is to determine the opinion of veterinary services about the possible causes of these new infections.

## Methods

### Study area

Colombia is a tropical and agro-pastoral country located in the northwestern region of South America and has a continental area of 1,141,748 km^2^. The cattle population is estimated at approximately 22.7 million head, half of which are dairy cattle, 30% of which are beef cattle, and 20% of which are dual purpose (beef and milk). A substantial portion of Colombian livestock is reared under traditional husbandry practices in rural areas with little technology, and these herds are the basis of the family economy.

### Study design

A case-control study was carried out to compare newly diagnosed herds with herds that remained brucellosis-free. The case farms were defined as those with herds that became infected with the disease during the period 2015 to 2016, having been previously classified as brucellosis-free for at least three years within the framework the official control programme. The control farms were those with herds that had retained disease-free status for a period equal to or greater than three years. These farms were randomly matched with case farms based on herd size and location at the municipality level. For five cases, we were not able to include a control, so the final sample size was 98 cases and 93 controls. This number of farms allowed the detection of differences in exposure of between 40 and 60% (OR = 2.25) with a confidence level of 95% and a power of 80%. The sampling frame was obtained from the Instituto Agropecuario Colombiano (ICA).

### Questionnaire

A questionnaire was created to obtain information about the potential risk factors; this questionnaire was based on existing literature and contained questions that focused on various topics, including the bovine brucellosis health programme, the physical characteristics of the farm (presence of fences, barns and milking facilities), animal husbandry, grazing, biosecurity, wild animals, and animal movements. The questionnaire in Spanish and its English translation is shown in Additional file 1: (Questionnaire for the study of bovine brucellosis in Colombia: Electronic Supplementary Material).

The questionnaire was evaluated in two stages; first, through a meeting with a panel of Colombian experts on the subject and, after that, by the veterinary officers of each affected region. Before answering the questionnaire, all farmers were informed that answering the questions was voluntary and that they did not have to answer all of the questions. All data were anonymously analysed. The feedback to the farmers was sent by the central veterinary service, which reported the results to field veterinarians, who in turn informed the farmers.

The questionnaires were completed by local veterinary officers through personal interviews with the farm owners. Additionally, following completion of the questionnaire and considering all the findings, the veterinary officer chose the most likely cause of re-introduction of the disease according to his/her opinion (only one cause). All variables obtained through the epidemiological questionnaire were categorical.

The information obtained via this questionnaire was complemented with other data, such as farm identification, census, vaccination status, location, and the brucellosis status of the interviewed farm and related farms (neighbours or origins of replacement heifers and bulls), which were obtained from the ICA database. The data were recorded in a Microsoft Office Excel 2017 file.

### Statistical analysis

Bivariate analysis was performed with the cases and controls using the chi-square test. Variables with a *p*-value of ≤0.20 were retained and included in the logistic regression analysis. In the case of collinearity among independent variables evaluated with the generalized variance inflation factors (GVIF) using a covariance matrix, the variable with the most biological sense was retained.

A stepwise logistic regression model was applied to the selected variables using forward selection. The analysis was started with an initial model without explanatory variables, and variables were introduced one by one, selecting those that best defined the dependent variable and gradually removing variables with no statistical significance (*p*-value > 0.05) in the model. The models were compared by the Akaike information criterion (AIC). All statistical analyses were performed using R Project software [18].

## Results

During the last two years, 98 previously brucellosis-free farms were infected and included in the study. These farms were located in fifteen departments, representing 60% of the Colombian cattle census (Antioquia, Arauca, Bolivar, Boyacá, Caldas, Cesar, Córdoba, Cundinamarca, La Guajira, Nariño, Putumayo, Quindío, Risaralda, Santander and Tolima), and the 93 controls were from the same departments.

Nineteen binary categorical variables had a *p*-value of ≤0.20 when they were compared between the cases and controls (Table 1). These variables were included in the logistic regression model.

**Table 1:** Categorical variables included in the bivariate analysis (chi-squared test with *p* < 0.2). Controls: 93 farms that remained brucellosis-free for at least three years. Cases: 98 herds previously free of brucellosis that became infected

Variables	Control	Case	OR	95% CI		
				Lower	Upper	p-value
*Livestock type*						
Dairy^a^	68 (73.12%)	60 (61.22%)	1			
Beef	8 (8.60%)	22 (22.45%)	3.12	1.29	7.52	0.009
Dual purpose (beef and milk)	17 (18.28%)	16 (16.33%)	1.07	0.5	2.29	0.869
*Equipment shared with other farms*						
No^a^	81 (87.10%)	77 (78.57%)	1			
Yes	12 (12.90%)	21 (21.43%)	1.84	0.85	4	0.12
*Cleaning and disinfection of clinical equipment and clothing*						
Yes^a^	86 (92.47%)	85 (86.73%)	1			
No	7 (7.53%)	13 (13.27%)	1.88	0.72	4.94	0.197
*Presence of facilities to shelter animals*						
No^a^	41 (44.09%)	31 (31.63%)	1			
Yes	52 (55.91%)	67 (68.37%)	1.7	0.94	3.08	0.077
*Farm fencing*						
Completely^a^	91 (97.85%)	91 (92.86%)	1			
Partially	2 (2.15%)	7 (7.14%)	3.5	0.71	17.3	0.105
*Contact of the herd with other neighbouring farms*						
No^a^	78 (83.87%)	67 (68.37%)	1			
Yes	15 (16.13%)	31 (31.63%)	2.41	1.2	4.83	0.012
*Infected animals in the neighbouring herd*						
No^a^	7 (7.53%)	2 (2.04%)	1			
Yes	86 (92.47%)	96 (97.96%)	3.91	0.79	19.32	0.074
*Mixed farm (farms that have species other than buffaloes or cattle)*						
No^a^	80 (86.02%)	72 (73.47%)	1			
Yes	13 (13.98%)	26 (26.53%)	2.22	1.06	4.648	0.032
*Water access points shared with other farms* ^*b*^						
No farms or brucellosis-free farms^a^	59 (63.44%)	48 (48.98%)	1			
Non-brucellosis-free farms	34 (36.56%)	50 (51.02%)	1.81	1.01	3.22	0.045
*Presence of drainage from other farms*						
No^a^	58 (62.37%)	48 (48.98%)	1			
Yes	35 (37.63%)	50 (51.02%)	1.73	0.97	3.07	0.063
*Animal movement: Brucella status* ^*b*^ *of other herds at the destination*						
Brucellosis-free farms or no movement^a^	89 (95.70%)	87 (88.78%)	1			
Non-brucellosis-free farms	4 (4.30%)	11 (11.22%)	2.81	0.86	9.17	0.076
*Status of the origin of replacement animals*^*b*^ *(internal or brucellosis-free farms* vs *calves from non-brucellosis-free farms)*						
Brucellosis-free farms or internal^a^	85 (91.40%)	67 (68.37%)	1			
Non-brucellosis-free farms	8 (8.60%)	31 (31.63%)	4.92	2.12	11.39	< 0.001
*Internal replacement with calves from the same farm whose mothers were positive*						
No^a^	89 (95.70%)	78 (79.59%)	1			
Yes	4 (4.30%)	20 (20.41%)	5.71	1.87	17.41	0.001
*Introduction of heifers < 24 months old into the farm*						
No admittance^a^	79 (84.95%)	72 (73.47%)	1			
Admittance	14 (15.05%)	26 (26.53%)	2.04	0.99	4.2	0.052
*Number of cattle introduced into the farm (per year)*						
None^a^	66 (70.97%)	53 (54.08%)	1			
Ten or fewer	19 (20.43%)	23 (23.47%)	1.51	0.74	3.06	0.255
Between eleven and thirty	6 (6.45%)	10 (10.20%)	2.08	0.71	6.08	0.178
More than thirty	2 (2.15%)	12 (12.25%)	7.47	1.6	34.85	0.004
*Infection status of the origin of the cattle* ^*b*^ *(of all ages)*						
Tested negative for *Brucella* or brucellosis-free farm^a^	71 (76.34%)	58 (59.18%)	1			
Not tested, unknown status of the farm, positive herds	22 (23.66%)	40 (40.82%)	2.23	1.19	4.16	0.012
*Introduction of other domestic species into the farm*						
None^a^	88 (94.63%)	85 (86.74%)	1			
More than twenty	0 (0.0%)	3 (3.06%)	7.25	0.37	142.37	0.13
Less than or equal to twenty	5 (5.37%)	10 (10.20%)	2.07	0.68	6.31	0.194
*Brucella status* ^*b*^ *of the origin of the bulls*						
Artificial insemination^a^	36 (38.71%)	34 (34.69%)	1			
Free herds	44 (47.31%)	16 (16.33%)	0.39	0.18	0.81	0.011
Unknown/positive status	13 (13.98%)	48 (48.98%)	3.91	1.81	8.46	< 0.001
*Access of dogs and cats to/contact with aborted foetuses and reproductive discharges*						
No (or no dogs)^a^	66 (70.97%)	59 (60.20%)	1			
Yes	27 (29.03%)	39 (39.80%)	1.62	0.88	2.95	0.119

^a^Category considered as reference. Variables with more than two categories have been pairwise compared with this category

^b^The brucellosis status of other farms was obtained from the tests performed within the framework of the control programme

The significant variables in the bivariate analysis (*p* < 0.05) were the type of livestock, direct contact, indirect contact with neighbouring farms through water, infection status of the origin of replacement heifers and bulls, number of cattle introduced into the farm, and status of the mother (for internal replacements).

The final multivariable logistic model (Table 2) included three risk factors: Herds that bring in heifers bought from farms with an unknown brucellosis status had a higher risk of infection than those that obtain replacements from brucellosis-free herds or that replace from within (OR = 4.84, *p*-value: < 0.001). Beef cattle farms were more affected (OR = 3.61, p-value = 0.017) than dairy or dual-purpose cattle farms. Finally, farms that use bulls from farms with positive/unknown *Brucella* status had a higher risk (OR = 2.45, p-value = 0.037) than those that use artificial insemination (AI). Farms with natural breeding that use bulls from brucellosis-free farms were less affected (OR = 0.30, p-value = 0.004) than those that use AI.

**Table 2:** Risk factors associated with new brucellosis infections in cattle farms compared with farms that remained brucellosis-free for at least three years based on multiple logistic regression model

Variables	B (SE)	OR	95% CI OR	p-value
(Intercept)	−0.35 (0.26)	0.71	(0.43; 1.18)	0.184
Brucellosis-free farms or internal replacement		1		
Replacement from farms with unknown/positive brucellosis status	1.58 (0.47)	4.84	(1.92; 12.20)	< 0.001
Type: Dairy		1		
Type: Beef	1.28 (0.54)	3.61	(1.26; 10.35)	0.017
Type: Dual purpose (beef and milk)	0.18 (0.47)	1.20	(0.48; 2.99)	0.696
Artificial insemination		1		
*Brucella*-free status of the origin of the bulls	−1.21 (0.42)	0.30	(0.13; 0.69)	0.004
Positive/unknown *Brucella* status of the origin of the bulls	0.90 (0.43)	2.45	(1.06; 5.68)	0.037

*B* coefficient estimated by the model, *SE* standard error, *OR* odds ratio, *95% CI OR* confidence interval of the OR (lower limit of the 95% CI; upper limit of the 95% CI)

The questionnaire included a question asking the opinion of the veterinarians that did the interview about the causes of infection. Table 3 shows the most cited answers; the three most frequent were contact with other domestic species (18%), contamination through water or feed (15%) and the introduction of infected cattle (14%). For most of the cases (80%), it was assumed that the new infection was exogenous; the reasons proposed for re-infection of the other 20% included resurgence due to keeping animals diagnosed as positive (7%), the absence/deficiency of biosecurity on the farm (5%) and the re-circulation of the bacteria (8%). Only the introduction of infected animals coincided with the results of the model.

**Table 3:** Opinion of the veterinary officer that completed the questionnaire concerning the most likely cause of the introduction of the brucellosis infection into the cattle farms

Causes of new infection	%
Exogenous origin of the new infection	80
Contact with other domestic species	18
Contaminated water/feed sources	15
Introduction of infected animals	14
Admittance of animals that have been in contact with seropositive animals in the originating farm	10
Transhumance/pasture sharing	8
Contact with infected people	4
Contact with wild species	4
Artificial insemination or embryo transfer	3
Movement of animals without official control	2
Endogenous origin of the new infection	20
Recirculation	8
Maintenance of animals diagnosed as positive	7
Absence/deficiency of biosecurity on the farm	5

## Discussion

Bovine brucellosis is endemic in Colombia [17] and, over the last 20 years, has been subject to an official control programme based on the compulsory vaccination of all bovine and bubaline females between 3 and 8 months of age and on a voluntary programme of testing and removing positive animals. Despite this programme, the incidence of brucellosis has remained stable, with the percentage of positive farms and positive animals averaging 22–23% and 4.7–4.6%, respectively, between 2006 and 2012 [17]. One of the reasons for the lack of progress in brucellosis control is the detection of new infections in herds that had previously been brucellosis-free. Between January 2015 and December 2016, 98 farms became infected. In this case-control study, we determined the possible factors related to these new infections. When these farms were compared with 93 herds that remained brucellosis-free, three factors were statistically significant: the origin of replacement animals, type of animal (dairy, beef or dual purpose) and use of AI or natural mating.

One of the more important causes of the introduction of diseases into non-affected populations is the introduction of infected animals: The acquisition of replacement animals from farms with an unknown status or where brucellosis is present carries a significant risk of introducing the infection [12, 19]. In Colombia, the origin of replacement animals was also the most significant factor associated with the new infections, with an OR of 4.8 (32% of the herds that became infected had brought in animals from non-brucellosis-free farms, compared with 9% of the controls).

Beef cattle farms had a higher risk of becoming infected than farms with dairy or dual-purpose herds (OR = 3.6, using dairy cattle as the reference). In general, beef cattle producers are more reluctant than dairy producers to apply control measures because they do not appreciate the benefits deriving from a brucellosis-free herd: The disease does not affect prices, and they do not understand the importance and the cost of reproductive disorders. Additionally, animals are distributed over large, extensive grazing areas that are usually difficult to access, where the control measures are more difficult to apply, and the animals are prone to greater contact with uncontrolled contaminated sources.

In contrast, dairy cattle producers are fully aware of the loss of production due to abortions and infertility and the subsequent important milk losses [20]. Furthermore, brucellosis-free herds can obtain more competitive prices for milk than non-certificated farms, because brucellosis is a concern for the milk industry and customers pay a bonus to brucellosis-free herds. Thus, dairy farmers are more prone to adopt the official control programme and to apply prevention and control measures more strictly than beef producers do.

The third variable that was significantly different between the cases and controls was the origin of the bulls. As expected, the entry of bulls from herds with a positive or unknown brucellosis status was a risk factor for the introduction of the infection in previously brucellosis-free herds. Surprisingly, the use of AI carries a higher risk than natural mating with bulls derived from herds certified as brucellosis-free (*p* = 0.004); these differences are probably due to the common practice on some farms of using fresh semen obtained from bulls on neighbouring farms. The use of artificial insemination with refrigerated semen obtained from a farm’s own bulls is a common practice in Colombia, and some farmers sell semen to neighbouring farms without any previous sanitary control. In our opinion, this lack of sanitary control is the reason that farms using AI have a higher risk than those that use natural mating with bulls from brucellosis-free herds. Unfortunately, we could not differentiate between insemination with refrigerated semen and insemination with frozen semen. Frozen semen comes from insemination centres certified as brucellosis-free by veterinary services.

In this study, the role of neighbouring herds was not observed except for the use of fresh semen, as mentioned above. Brucella infection has been associated with the spread of infection through direct contact with cattle from neighbouring herds and through the exchange of bulls between farms for mating [21]. Indirect contact with neighbouring farms, such as through shared drinking water access points and grazing lands, also constitutes a risk for the transmission of brucellosis between herds [7, 21].

The presence of mixed farms, including interactions with small ruminants, has also been associated with high prevalence of brucellosis [22] and with a resurgence of the disease in cattle herds [14]. A relationship between these species and brucellosis was not observed in our study, despite traditional husbandry practices in Colombia, including rearing different species in the same habitat.

The results of this study contribute to understanding new infections of previously infection-free herds and may help decision makers in Colombia and other tropical countries with a similar situation to improve control strategies for bovine brucellosis.

The factors described here are well known. Indeed, veterinarians recommend the need to introduce only animals from brucellosis-free herds and to require a brucellosis-free certification for semen, but veterinary officers believe that many farmers do not comply with such recommendations. Consequently, it is important to emphasize to cattle owners the risk involved in the introduction of animals and semen from farms of unknown health status. Furthermore, control measures for the introduction of animals into brucellosis-free herds, including a quarantine of newly acquired animals to assure that these animals are negative, should be strictly applied.

As the prevalence of Brucella is relatively low, culling positive animals should be mandatory and reinforced with an educational programme for farmers to highlight the importance of brucellosis as a zoonotic disease [17, 21]. This campaign should also include the importance of biosecurity measures to reduce the spread of brucellosis between and within herds.

Finally, when the risk factors were compared with the veterinary officers’ opinions, there was substantial agreement on the consequences of introducing infected animals into the herd but not on other significant variables such as the role of artificial insemination with semen from non-controlled herds. Additionally, other variables that can also play a role in reintroducing brucellosis were cited. This analysis reflects the fact that there are distinct perceptions of possible causes for the introduction of brucellosis into herds and highlights a lack of knowledge regarding some of these causes.

## Conclusions

Three risk factors for the re-introduction of brucellosis into previously brucellosis-free herds were identified: the introduction of replacement animals and bulls from farms with unknown or positive brucellosis status, the use of AI with semen from neighbouring non-brucellosis-negative herds and the identity of a herd as a beef herd.

Governments should reformulate strategies to extend the programme to a larger number of farms and to make the programme mandatory in the medium term. In addition to vaccination and test-and-slaughter strategies, the programme needs to include an awareness campaign that focuses on the cost of the disease, even for beef producers, and on the importance of avoiding risk factors such as the introduction of replacement animals or the use of fresh semen from uncontrolled herds.

## Additional file

Additional file 1: Questionnaire for the sanitation study of bovine brucellosis in Colombia (DOCX 127 kb)

## Abbreviations

AI: Artificial insemination
AIC: Akaike information criterion
ICA: Instituto Agropecuario Colombiano
VIF: Variance inflation factor

## References

[1] Moreno E (2014). Retrospective and prospective perspectives on zoonotic brucellosis. Front Microbiol.

[2] Heller MC, Watson JL, Blanchard MT, Jackson KA, Stott JL, Tsolis RM (2012). Characterization of *Brucella abortus* infection of bovine monocyte-derived dendritic cells. Vet Immunol Immunopathol.

[3] de Alencar Mota ALA, Ferreira F, Ferreira Neto JS, Dias RA, Amaku M, Hildebrand Grisi-Filho JH (2016). Large-scale study of herd-level risk factors for bovine brucellosis in Brazil. Acta Trop.

[4] Carvalho Neta AV, Mol JP, Xavier MN, Paixão TA, Lage AP, Santos RL (2010). Pathogenesis of bovine brucellosis. Vet J.

[5] O’Grady D, Byrne W, Kelleher P, O’Callaghan H, Kenny K, Heneghan T (2014). A comparative assessment of culture and serology in the diagnosis of brucellosis in dairy cattle. Vet J.

[6] Ducrotoy MJ, Bertu WJ, Ocholi RA, Gusi AM, Bryssinckx W, Welburn S (2014). Brucellosis as an emerging threat in developing economies: lessons from Nigeria. PLoS Negl Trop Dis.

[7] Terefe Y, Girma S, Mekonnen N, Asrade B (2017). Brucellosis and associated risk factors in dairy cattle of eastern Ethiopia. Trop Anim Health Prod.

[8] Godfroid J, Al Dahouk S, Pappas G, Roth F, Matope G, Muma J (2013). A “one health” surveillance and control of brucellosis in developing countries: moving away from improvisation. Comp Immunol Microbiol Infect Dis.

[9] Ragan V, Vroegindewey G, Babcock S (2013). International standards for brucellosis prevention and management. Rev Sci Tech.

[10] Gibbs J, Bercovich Z, Fuquay JE, Fox PF, McSweeney PLH (2011). Diseases of dairy animals. Infectious diseases: brucellosis. Reference module in food science. Encyclopedia of dairy sciences.

[11] De Figueiredo P, Ficht TA, Rice-Ficht A, Rossetti CA, Adams LG (2015). Pathogenesis and immunobiology of brucellosis: review of brucella–host interactions. Am J Pathol.

[12] Stringer LA, Guitian FJ, Abernethy DA, Honhold NH, Menzies FD (2008). Risk associated with animals moved from herds infected with brucellosis in Northern Ireland. Prev Vet Med.

[13] Mekonnen H, Kalayou S, Kyule M (2010). Serological survey of bovine brucellosis in Barka and arado breeds (*Bos indicus*) of Western Tigray. Ethiopia Prev Vet Med.

[14] Ducrotoy M, Bertu WJ, Matope G, Cadmus S, Conde-Álvarez R, Gusi AM (2017). Brucellosis in sub-Saharan Africa: current challenges for management, diagnosis and control. Acta Trop.

[15] Hegazy YM, Molina-Flores B, Shafik H, Ridler AL, Guitian FJ (2011). Ruminant brucellosis in upper Egypt (2005-2008). Prev Vet Med..

[16] Guarnizo PL (2014). Estudio descriptivo de la presentación de brucelosis humana en Colombia desde 2000 hasta 2012. Revista de Medicina Veterinaria.

[17] Cárdenas L, Melo O, Casal J. Evolution of bovine brucellosis in Colombia over a 7-year period (2006–2012). Trop Anim Health Prod. 2017:1–11. 10.1007/s11250-017-1395-4. 10.1007/s11250-017-1395-4 28905264

[18] R Core Team. R. A language and environment for statistical computing. Vienna, Austria: R Foundation for Statistical Computing; 2017. http://www.R-project.org/. Accessed 02 Oct 2017.

[19] Musallam I, Abo-Shehada M, Omar M, Guitian J (2015). Cross-sectional study of brucellosis in Jordan: prevalence, risk factors and spatial distribution in small ruminants and cattle. Prev Vet Med..

[20] Roth F, Zinsstag J, Orkhon D, Chimed-Ochir G, Hutton G, Cosivi O, Carrin G, Otte J (2003). Human health benefits from livestock vaccination for brucellosis: case study. Bull World Health Organ.

[21] Alhaji NB, Wungak YS, Bertu WJ (2016). Serological survey of bovine brucellosis in Fulani nomadic cattle breeds (*Bos indicus*) of north-Central Nigeria: potential risk factors and zoonotic implications. Acta Trop.

[22] Zamri-Saad M, Kamarudin MI (2016). Control of animal brucellosis: the Malaysian experience. Asian Pac J Trop Med.

