# Supplementary material for: Risk factors for new bovine brucellosis infections in Colombian herds
Source: BMC Vet Res. 2019 Mar 7;15:81. doi: 10.1186/s12917-019-1825-9 (PMC6404332; doi:10.1186/s12917-019-1825-9)
Supplement: Supplementary file 1 — Questionnaire for the sanitation study of bovine brucellosis in Colombia (DOCX 127 kb) [file 12917_2019_1825_MOESM1_ESM.docx]

**S1. Electronic Supplementary Material**

| **CODIGO PREDIO:**  ***FARM CORDE:*** | | | |
| --- | --- | --- | --- |
| Fecha: *Date:* | ***D*** | ***M*** | ***Y*** |

**Encuesta de estudio sanitario de la**

**Brucelosis bovina en Colombia**

***Survey of the sanitary study of bovine***

***Brucelosis in Colombia***

**Tipo de encuesta:** Caso________ Control_________ *Survey type: Case________ Control_________*

**Información general del predio: *General information of the farm:***

| Departamento: *Department:* | Municipio: *Town:* | Vereda: *Road/Route:* | |
| --- | --- | --- | --- |
|  |  |  | |
| Nombre del Predio: *Name of the farm:* | Nombre del propietario: *Name of the owner:* | | |
|  |  | | |
| Teléfono de contacto: *Contact telephone:* | Email de contacto: *Email contact* | | |
|  |  |  |  |
| Ubicación: *Location:* | Latitud: *Latitude:* | Longitud: *Longitude:* |  |
|  |  |  |  |
| Extensión del predio: *Area of the farm:* | Existencia de otros propietarios en el predio: *Existence of other owners on the same farm:* | | |
|  | SI (¿Cuántos?) *YES (¿How many?)* NO | | |

**Programa sanitario de Brucelosis bovina**

***A. Sanitary program of bovine Brucelosis***

*Certificación como predio libre Certification as a free farm*

1. Fecha de obtención (*última certificación*): ___**DD**______ /____**MM**______ /____**AAAA**______

Estado actual: Libre vigente___________ Infectado en saneamiento__________ Infectado no en saneamiento__________

*1. Date of certification (last certification): D____/M____Y____ Current state: Currently free_________ Infected, in a control program Infected, not in a control program __________*

2. La empresa responsable del muestreo de la certificación de predio libre ha sido siempre la misma? (*Considerar los últimos 5* *años*) Sí____ No____

*2. The company responsible of the sampling has been always the same? (Considering the last 5 years) Yes _____ No _____*

3. ¿En caso de que SI, el veterinario inspector de la empresa ha sido siempre el mismo? (máximo ha cambiado una vez) Sí___ No___

*3. If YES, has performed the sampling always the same veterinarian or two vets during the whole period? Yes _____ No _____*

4. En caso de que NO, indicar las distintas empresas que han intervenido:

*4. If NOT, indicate the different companies that have participated:*

| **Nombre de la empresa *Name of the company*** | **Realiza otros servicios veterinarios de campo (SI/NO) *They perform* *other veterinary services (YES/NO)*** | **Periodo *Period*** | |
| --- | --- | --- | --- |
|  |  | **Año inicio**  ***First year*** | **Año final**  ***Last year*** |
|  |  |  |  |
|  |  |  |  |
|  |  |  |  |

5. ¿El médico veterinario que presta asesoría técnica en su predio, atiende otras explotaciones con signos compatibles con Brucelosis bovina? Sí___ No___ No se____

*5. The vet who gives technical assessment in your farm, does he/she attend to other farms with signs compatible with bovine Brucelosis? Yes _____ No _____ I don´t know _____*

6. Es exclusivo el personal que labora en el predio, o presta servicio técnico en otras explotaciones? Sí______ No______ No se________

*6. The personnel that works on the farm only work on that one (or they give technical service at other farms? Yes _____ No _____ I don´t know _____*

**B. Características del predio**

***B. Characteristics of the farm***

1. Aptitud ganadera (marque una “X” en la actividad)

*1. Livestock activity (mark the activity with an “X”)*

| Leche  *Dairy* | Carne  *Beef* | Doble propósito *Dual purpose (beef and milk)* |
| --- | --- | --- |
|  |  |  |

2. Número de animales en el predio (bovinos y/o búfalos)

*2. Number of animals on the farm (bovines and/or buffaloes)*

| **TERNERAS < 1 AÑO**  ***Calves <1-YEAR-OLD*** | **HEMBRAS 1 - 2 AÑOS**  ***COWS 1-2 YEARS OLD*** | **HEMBRAS 2 - 3 AÑOS**  ***COWS 2-3 YEARS OLD*** | **HEMBRAS > 3 AÑOS**  ***COWS > 3 YEARS OLD*** | **TERNEROS < 1 AÑO**  ***CALFS <1-YEAR-OLD*** | **MACHOS 1 - 2 AÑOS**  ***BULLS 1-2 YEARS OLD*** | **MACHOS 2 - 3 AÑOS**  ***BULLS 2-3 YEARS OLD*** | **MACHOS > 3 AÑOS**  ***BULLS >3 YEARS OLD*** | **TOTAL, BOVINOS/ BUFALINOS**  ***BOVINE/BUFFALO TOTAL*** |
| --- | --- | --- | --- | --- | --- | --- | --- | --- |
|  |  |  |  |  |  |  |  |  |
|  |  |  |  |  |  |  |  |  |

3. Número de animales de otras especies en el predio (incluye todas las edades)

*3. Number of animals of other species on the farm (includes all ages)*

| **Especie**  ***Species*** | **NO** | **SI: Cantidad**  ***YES: Quantity*** |
| --- | --- | --- |
| Ovinos  *Ovine* |  |  |
| Caprinos  *Goat* |  |  |
| Porcinos  *Porcine* |  |  |
| Equinos  *Equine* |  |  |
| Perros  *Dogs* |  |  |
| Gatos  *Cats* |  |  |

4. Las otras especies indicadas en el cuadro anterior, tienen contacto directo con los bovinos y/o búfalos del predio? No______, Si accidentalmente _______ ¿Cuáles? ________, Si normalmente _______ ¿Cuáles? ________

*4. These other species have direct contact with the bovines and/or buffaloes of the farm? No _____, Yes, accidentally _____ (¿Which ones?) _____ Yes, usually _____ (Which ones?) _____*

5. Existen o transitan por el predio animales silvestres? Si____ No____. De ser SI, ¿Cuáles? _____

*5. Are wild animals in the farm? Yes _____ No _____. If the answer is YES, Which ones? _____*

6. Instalaciones (Tipo de explotación)

*6. Facilities (Type of farm)*

a) Indique con cuales de las siguientes instalaciones cuenta su predio:

Cercas_____, Corrales______, Establos_____ Salas de ordeño______, Otros ¿Cuáles? _______________

*a) Indicate which of the following installations your farm has:*

*Fences _____, Pens _____, Stables _____ Milking rooms _____, Others, Which ones? ______________*

b) Indique que tipo de predio posee, siendo predio cerrado aquel que se abastece por sí mismo de animales, predio abierto aquel que compra animales y predio semi abierto aquel que compra animales ocasionalmente: Predio cerrado______, predio abierto______, predio semi abierto______

*b) Indicate the type of farm (closed farm: is farm performing self-replacement; open farm: Farm that purchase heifers): Closed farm _____, Open farm _____, Semi-open farm _____*

c) Especifique el número de núcleos de su predio, es decir la cantidad de grupos de animales de la misma especie (bovinos y/o búfalos) que comparten características etarias y/o productivas. Ejemplo: cría, levante, vacas secas, entre otros. Número de núcleos del predio: ________

*c) Specify the number of nuclei in your farm, that is, the number of animal groups of the same species (bovines and/or buffaloes) that share age and/or productive characteristics. For example: newborn, yearling, dry cows, among others. Number of nuclei in the farm: __________*

7. Tipo de manejo:

*7. Type of management:*

| Intensivo *Intensive* | Semi-intensivo *Semi-intensive* | Extensivo *Extensive* |
| --- | --- | --- |

8. Manejo de la leche:

*8. Milk management:*

| Ordeño manual  *Manual milking* | Ordeño mecánico  *Mechanical milking* | Sin ordeño  *No milking* |
| --- | --- | --- |

9. Indique el método de reproducción que emplea en su predio, especifique el porcentaje del uso y la procedencia del mismo.

*9. Indicate the method of reproduction used, specify the percent and the origin of the semen.*

| **Porcentaje de uso (%)**  ***Percent of use (%)*** | **Método de reproducción**  ***Method of reproduction*** | **Procedencia**  ***Origin*** |
| --- | --- | --- |
|  | Monta natural  *Natural mating* |  |
|  | Inseminación artificial (semen registrado)  *Artificial insemination (semen registered)* |  |
|  | Inseminación artificial (semen no registrado)  *Artificial insemination (unregistered semen)* |  |
|  | Transferencia de embriones (registrados)  *Embryo transfer (registered)* |  |
|  | Transferencia de embriones (no registrados)  *Embryo transfer (unregistered)* |  |

**C. Pastoreo**

***C. Pasture***

1. Área total de pastoreo (en el predio): ______ (Ha)

*1. Total, pasture área (on the farm): _____ (Ha)*

2. ¿Realiza trashumancia? Sí____ No_____

*2. They do transhumance? Yes _____ No _____*

3. En caso afirmativo: Distancia recorrida (Km): _________ A otra vereda_______ A otro municipio________

Predio de destino: _____________ Es predio libre de Brucelosis bovina? Si____ No___

*3. If YES: Distance covered (Km) _______ To/On another village _____ To/On another municipality _______*

*Farm of destination: __________ Is the farm free of bovine Brucelosis? Yes _____ No _____*

4. ¿Los animales del predio comparten áreas de pastoreo con animales de otros predios susceptibles? Sí______ No_______

*4. Do the animals of the farm share pasture with animals from other susceptible farms? Yes _____ No _____*

5. ¿Los animales del predio tienen contacto con animales de otros predios vecinos? Si___ No____.

¿En caso afirmativo, con cuál especie? Bovina _____ Bufalina ____ Caprina _____ Ovina____ Equina_____ Porcina_____ Perros_____ Otras ¿Cuáles? _________________________________

*5. Have the animals of the farm contact with animals of neighboring farms? Yes _____ No _____.*

*If YES, with which species? Bovine _____ Buffalo _____ Goat _____ Ovine _____ Equine _____ Porcine _____ Dogs _____ Others: Which ones? ____________________________________*

6. Listado de predios de contacto a menos de 2km (vecinos) y su calificación sanitaria

*6. List the farms located at less than 2km (neighbors) and their sanitary qualification*

| **Nombre del predio**  ***Name of the farm*** | **Especies animales**  ***Animal species*** | **Número de animales (aprox)**  ***Number of animals (approx.)*** | **Estado sanitario: Predio libre de Brucelosis bovina SI/NO**  ***Sanitary state: free of bovine Brucelosis YES/NO*** |
| --- | --- | --- | --- |
|  |  |  |  |
|  |  |  |  |
|  |  |  |  |

7. Indique las fuentes de agua que tienen acceso los animales del predio:

Nacimientos en el predio______ ríos/quebradas_____ Pozos_____ Aljibes_____ Fuente de agua proveniente de otro predio_____ Acueducto municipal________

*7. Indicate the sources of water of the farm:*

*Spring on the farm _____ Rivers/gullies _____ Wells _____ Tank_____ Source of water coming from another farm _____ Municipal aqueduct _____*

8. ¿Comparte vertientes de agua o puntos de agua con otros predios? Si_____ No____.

En caso de que SI, ¿Están esos predios certificados como libres de Brucelosis bovina? Sí____ No____ No Se_______

*8. Do you share water springs or points of water with other farms? Yes _____ No _____*

*If YES, are those farms certified as being free of bovine Brucelosis? Yes _____ No _____ I don´t know _____*

9. ¿Recibe drenaje o desagüe de otros predios?  Sí____ No_____

*9. Do you receive drainage from other farms? Yes _____ No _____*

10. ¿Su predio drena o desagua en otros predios? Sí____ No_____

*10. Does your farm drain into other farms? Yes _____ No _____*

**D. Bioseguridad**

***D. Biosafety***

1. ¿El predio está cercado? Completo_____ Parcial _____

*1. Is the farm closed? Completely _____Partially _____*

2. ¿Su predio comparte maquinaria (Tractor, camión de transporte, etc.) con otros predios? Si___ No____

*2. Does your farm share machinery (Tractor, transport truck, etc.) with other farms? Yes _____*

*No _____*

3. ¿Realiza aislamiento (separación) inmediata y efectiva de los animales seropositivos, sospechosos o con nexos epidemiológicos reproductivos? Sí___ No____

En caso de que SI, describa el procedimiento sanitario. *(Responder al reverso de la hoja)*

*3. Do you carry out immediate and effective isolation (separation) of the sero-positive animals, suspicious animals, or those with epidemiological links? Yes _____ No _____*

*If YES, describe the sanitary procedure. (Respond on the back of this sheet)*

4. Si su predio es considerado infectado por *Brucella abortus* sacrifica los animales positivos? Sí_____ No____

En caso afirmativo, indicar tiempo de permanencia de los animales positivos desde el diagnostico hasta el sacrificio (días) ________

*4. If your farm is infected by Brucella abortus, do you slaughter the test positive animals? Yes _____ No _____*

*If YES, indicate the time lapsed between test and removal (days) _____*

5. ¿En los últimos dos diagnósticos serológicos de laboratorio en procura de recertificación, se ha dejado algún animal sin examinar? Si____ No____ En caso afirmativo, indicar:

Cuántos en el penúltimo muestreo_______ Cuántos en el último muestreo_______

*5. In the last two serological diagnose rounds, all animals have been sampled? Yes _____ No _____ If NOT, indicate:*

*How many in the next-to-the-last round _____ How many in the last sampling _____*

6. ¿Son lavados y desinfectados equipos, materiales e indumentaria que han estado en contacto en la manipulación de animales, al menos una vez por semana? Sí_____ No____

*6. ¿Are the equipment, materials and clothing that have been in contact with the manipulation of animals been washed and disinfected at least once a week? Yes _____ No _____*

7. ¿Es removido el estiércol en el lugar de alojamiento de los animales diariamente? Sí_____ No____

*7. Manure of the animals’ facilities is removed daily? Yes _____ No _____*

8. ¿Puede contaminarse el alimento (forraje, heno) y agua con heces y orina de animales del predio? Sí_____ No____

*8. ¿Can the feed (forage, hay) and water be contaminated with feces and urine of the farm’s animals? Yes _____ No _____*

**E. Factores ambientales y contacto con animales silvestres**

***E. Environmental factors and contact with wild animals***

1. En caso de existir contacto con animales silvestres, indique la distancia del área del bosque a los bovinos o búfalos de su predio __________ (Km)

*1. In the event of contact with wild animals, indicate the distance between the forest and the bovines or buffaloes of your farm. _____ (Km)*

**F. Aspectos sanitarios**

***F. Sanitary aspects***

1. El origen de la reposición de bovinos y/o bufalinos es:

Interno ____ Externo___. En el caso de origen externo, ¿Proceden de predio libre de Brucelosis bovina? Sí____ No____

*1. The origin of the replacement of the bovines and/or buffaloes is:*

*Internal _____ External _____. In case of external origin, do they come from a farm free of bovine Brucelosis? Yes _____ No _____*

2. ¿Alguno de los animales de reposición resultó positivo? Sí______ No______

*2. Was some of the replacement animals positive? Yes _____ No _____*

3. ¿Ingresan al predio hembras menores de 24 meses? Si____ No____, En caso afirmativo, procedentes de: predio libre_____ predio no certificado como libre con terneras vacunadas______ ó

Con resultados negativos a *Brucella abortus _*_____

*3. Did you purchase heifers less than 24 months old? Yes _____ No _____ If YES, where did they come from: Disease-free farm _____ Uncertified farm as being free, with vaccinated calfs _____ or with negative results for Brucella abortus _____*

4. ¿Ha hecho reposición con hijas de vacas de su propio predio que han resultado positivas? Sí____ No____

*4. Have you done replacements with daughters of positive cows of your own farm? Yes _____ No _____*

5. ¿Alguna de las novillas de reposición es hija de una vaca que ha dado resultado positivo, de manera que podía haber sido positiva durante la gestación o en el año siguiente? Sí____ No____

*5. Have any of the replacement heifers been the daughter of a positive cow that that could have been positive during pregnancy or during the following year? Yes _____ No _____*

6. Tasa de abortos ó de nacidos débiles que mueren en la primera semana (*último año*) _______%

*6. Abortion rate or rate of weak calves that die within the first weak (last year) _____%.*

7. ¿Describa qué se hace con los fetos abortados y residuos de descargas reproductivas? *(Responder al reverso de la hoja)* ¿Tienen acceso a ellos perros o gatos? Sí____ No____

*7. Describe what do you do with the aborted fetuses and reproductive discharge residue. (Respond on the back of this sheet) Do dogs or cats have access to them? Yes _____ No _____*

8. Se realiza aislamiento de las hembras próximas al parto Sí____ No____

*8. Cows are isolated before calving? Yes _____ No _____*

9. Tipo de vacuna utilizada frente a Brucelosis bovina: Cepa 19_____ RB51_______ Una y otra________

*9. Type of vaccine used for bovine Brucelosis: Strain 19 _____ RB51 _____ Both _____*

10. ¿Hace revacunación (únicamente con RB51)? En caso de que Si, indicar la edad ___________

*10. Do you re-vaccinate animals (only with RB51)? If YES, indicate the age. ______*

11. ¿Quién realiza la vacunación en el predio? Vacunador oficial________ Veterinario asistente técnico/Veterinario OIA_________

*11. Who performs the vaccinations on the farm? Official vaccinator _____Veterinarian /OIA veterinarian _____*

12. Estimación de la tasa total de cobertura vacunal en el predio sobre el censo de hembras en edad de vacunación (en %) *(últimos 2 años)* ______

*12. Rate of vaccine coverage on the farm related to the census of cows in vaccination age (in %) (last 2 years) _____*

13. ¿Se tiene conocimiento de infección confirmada por *Brucella abortus* entre las personas que frecuentan o viven en el predio? Sí____ No____ Nose_____

*13. Do you know if there is any confirmed infection of Brucella abortus among the people who are frequently on or live on the farm? Yes _____ No _____ I don´t know _____*

**CONCLUSIONES DEL ENCUESTADOR SOBRE EL POSIBLE ORIGEN (ÚNICAMENTE PREDIOS CASO)**

***CONCLUSIONS OF THE SURVEY-TAKER REGARDING THE POSSIBLE ORIGIN (ONLY CASES)***

_____ Convivencia con otras especies domésticas/*Contact with other domestic species*

_____ Convivencia con otras especies silvestres/*Contact with wild species*

_____ Introducción de animales infectados *Introduction of infected animals*

_____ Uso de material genético infectado (inseminación artificial o transferencia de embriones) /*Artificial insemination or embryo transfer*

_____ Ingreso de animales seronegativos contacto de seropositivos/ *Admittance of animals that have been in contact with seropositive animals in the origin farm*

_____ No sacrificio de positivos/ *Maintenance of animals diagnosed as positive*

_____ Fuentes de alimentación o aguas contaminadas/ *Water/feed sources contaminated*

_____ Ausencia/deficiencia de bioseguridad en la granja/ *Absence / deficiency of biosecurity in the farm*

_____ Trashumancia/pastos comunales/ *Transhumance/pasture sharing*

_____ Contacto humano portador (infectado)/ *Contact with infected people*

_____ Recirculación/ *Recirculation*

_____ Traslado/movilización de animales sin documento oficial*/ Movement of animals without official control*

De lo anterior se concluye que el origen más probable de la infección sea: Exógeno____ Endógeno____

*From the above, it is concluded that the most probable origin of the infection is: Exogenous _____ Endogenous _____*

______________________________________________________

Nombre y firme de veterinario oficial responsable -ICA

*Name and signature official veterinarian in charge - ICA*

**G. Ingreso de animales**

***G. Entrance of animals***

1. bovinos y/o bufalinos 1. Bovines and/or buffaloes

| **DPTO /MUNICIPIO**  ***DEPT/MUNICIPALITY*** | **PREDIO (Nombre y No.)**  ***FARM (Name and Number)*** | **FECHA**  ***DATE*** | **TERNERAS < 1 AÑO**  ***FEMALE CALFS < 1 YEAR*** | **HEMBRAS 1 - 2 AÑOS**  ***COWS 1-2 YEARS*** | **HEMBRAS 2 - 3 AÑOS**  ***COWS 2-3 YEARS*** | **HEMBRAS > 3 AÑOS**  ***COWS > 3 YEARS*** | **TERNEROS < 1 AÑO**  ***MALE CALFS < 1 YEAR*** | **MACHOS 1 - 2 AÑOS**  ***BULLS 1-2 YEARS*** | **MACHOS 2 - 3 AÑOS**  ***BULLS 2-3 YEARS*** | **MACHOS > 3 AÑOS**  ***BULLS > 3 YEARS*** | **TOTAL BOVINOS**  ***TOTAL BOVINES*** | **PRUEBAS SEROLÓGICAS**  ***SEROLOGICAL TESTS*** | **RESULTADO A LAS PRUEBAS**  ***TESTS RESULTS*** |
| --- | --- | --- | --- | --- | --- | --- | --- | --- | --- | --- | --- | --- | --- |
|  |  |  |  |  |  |  |  |  |  |  |  |  |  |
|  |  |  |  |  |  |  |  |  |  |  |  |  |  |
|  |  |  |  |  |  |  |  |  |  |  |  |  |  |
|  |  |  |  |  |  |  |  |  |  |  |  |  |  |
|  |  |  |  |  |  |  |  |  |  |  |  |  |  |
|  |  |  |  |  |  |  |  |  |  |  |  |  |  |
|  |  |  |  |  |  |  |  |  |  |  |  |  |  |
|  |  |  |  |  |  |  |  |  |  |  |  |  |  |
|  |  |  |  |  |  |  |  |  |  |  |  |  |  |
|  |  |  |  |  |  |  |  |  |  |  |  |  |  |
|  |  |  |  |  |  |  |  |  |  |  |  |  |  |
|  |  |  |  |  |  |  |  |  |  |  |  |  |  |

2. Otras especies de animales

*2. Other animal species*

| **DPTO/MPIO**  ***DEPT/MUNICIPALITY*** | **PREDIO (Nombre y No.)**  ***FARM (Name and Number*** | **EL PREDIO ORIGEN ES LIBRE DE BRUCELOSIS BOVINA? SI/NO**  ***IS THE FARM FREE OF BOVINE BRUCELOSIS? YES/NO*** | **FECHA DE INGRESO**  ***ENTRY DATE*** | **OVINOS**  ***OVINES*** | **CAPRINOS**  ***GOATS*** | **EQUINOS**  ***EQUINE*S** | **PORCINOS**  ***PORCINES*** | **TOTAL ANIMALES**  ***TOTAL ANIMALS*** | **PRUEBAS SEROLÓGICAS**  ***SEROLOGICAL TESTS*** | **RESULTADO A LAS PRUEBAS**  ***TESTS RESULTS*** |
| --- | --- | --- | --- | --- | --- | --- | --- | --- | --- | --- |
|  |  |  |  |  |  |  |  |  |  |  |
|  |  |  |  |  |  |  |  |  |  |  |
|  |  |  |  |  |  |  |  |  |  |  |
|  |  |  |  |  |  |  |  |  |  |  |
|  |  |  |  |  |  |  |  |  |  |  |
|  |  |  |  |  |  |  |  |  |  |  |
|  |  |  |  |  |  |  |  |  |  |  |
|  |  |  |  |  |  |  |  |  |  |  |
|  |  |  |  |  |  |  |  |  |  |  |
|  |  |  |  |  |  |  |  |  |  |  |
|  |  |  |  |  |  |  |  |  |  |  |
|  |  |  |  |  |  |  |  |  |  |  |
